# Upregulation of SOX2-activated lncRNA ANRIL promotes nasopharyngeal carcinoma cell growth

**DOI:** 10.1038/s41598-018-21708-z

**Published:** 2018-02-20

**Authors:** Jian-Hui Wu, Jian-Ming Tang, Jia Li, Xiong-Wen Li

**Affiliations:** 1 The Otolaryngological Department, Meizhou People’s Hospital, Meizhou, Guangdong, P.R. China; 2 State Key Laboratory of Oncogenes and Related Genes, Renji-Med X Clinical Stem Cell Research Center, Ren Ji Hospital, School of Medicine, Shanghai Jiao Tong University, Shanghai, P.R. China

## Abstract

Recent molecularly targeted approaches have gained advances in nasopharyngeal carcinoma treatment. However, the estimated five-year survival rate has not met the desired degree of improvement. Here, we report that upregulation of the expression of the SOX2-activated lncRNA ANRIL is involved in nasopharyngeal carcinoma. ANRIL has been found to be upregulated in clinical nasopharyngeal carcinoma. Using genetic approaches targeting ANRIL in nasopharyngeal carcinoma cells, we found that the knockdown of ANRIL inhibits cell proliferation *in vitro* and *in vivo*. Mechanistically, SOX2 binds with ANRIL and increases its RNA level, which upregulates β-catenin signalling, resulting in enhanced nasopharyngeal carcinoma tumourigenesis. Expression levels of ANRIL are positively correlated with SOX2 and β-catenin in clinical nasopharyngeal carcinoma samples. Our findings demonstrate that the SOX2-ANRIL-β-catenin axis plays a critical role in nasopharyngeal carcinoma proliferation and provide a potential therapeutic approach for nasopharyngeal carcinoma patients.

## Introduction

Nasopharyngeal carcinoma is one of the most common malignancies of the head and neck, and it has a multifactorial aetiology, presenting a distinct geographical distribution in occurrence, with high incidence rates in North Africa, Southeast Asia, and Southern China^1,2^. The estimated 5-year survival rate of nasopharyngeal carcinoma has not met the expectation for improvement despite recent advancements in molecularly targeted approaches in nasopharyngeal carcinoma treatment^3^. This lack of improvement in survival is due to distant metastases, concurrent radiotherapy and chemotherapy- treated nasopharyngeal carcinoma with distinctive clinical and pathologic features, and advanced stages^4,5^. The lack of targeted therapies and the poor prognosis for nasopharyngeal carcinoma patients have fostered a major effort to discover actionable molecular targets to treat patients with these tumours. Recent long non-coding RNAs (lncRNAs) have been recognized as playing critical roles in the progression of nasopharyngeal carcinoma^6–8^. However, the mechanisms by which lncRNAs mediate nasopharyngeal carcinoma tumourigenicity are not well known.

LncRNAs are a group of endogenous non-protein coding RNA molecules that are longer than 200 nt in length and that regulate gene expression. LncRNAs may promote the progression of breast cancer^9–11^. Increasing evidence has shown that many dysregulated lncRNAs play vital roles in tumourigenesis via transcriptional or post-transcriptional regulation^12–14^. Deregulation of lncRNAs has been found in multiple tumours, where lncRNAs can act as tumour suppressor genes or oncogenes. The lncRNA ANRIL (CDKN2B antisense RNA1) was initially identified from familial melanoma patients with neural system tumours, and it encodes a 3834-nt RNA consisting of 19 exons that is transcribed in the antisense orientation from the INK4B-ARF-INK4A gene cluster on chromosome 9p21. It contains three important tumour suppressors, p14ARF, p15INK4b, and p16INK4a^15,16^. Many studies have demonstrated that the upregulation of ANRIL plays an oncogenic role in a variety of tumours, including nasopharyngeal carcinoma^17^. Although ANRIL serves as a fatal oncogene in many cancers, the mechanism of ANRIL-driven nasopharyngeal carcinoma cell growth remains undefined.

The transcription factor SOX2 is widely recognized for its pivotal roles during mammalian embryogenesis^18^. Meanwhile, SOX2 has also been involved in controlling stem cell activity to regulate tumourigenesis, tumour proliferation, metastasis and progression in multiple types of cancer^19,20^. It has been reported that some transcription factors can bind to the promoters of lncRNAs to affect their transcription efficiency^21^. However, the interaction between SOX2 and ANRIL remains unclear.

In this study, we are the first to report that SOX2-induced ANRIL promotes nasopharyngeal carcinoma growth by promoting β-catenin. Furthermore, the SOX2-ANRIL-β-catenin axis plays important roles in nasopharyngeal carcinoma proliferation *in vivo*. We demonstrated that the expression level of ANRIL is upregulated in nasopharyngeal carcinoma tissues compared with that in normal tissues. The levels of ANRIL were positively correlated with those of SOX2 and β-catenin in nasopharyngeal carcinoma tissues. Therefore, our evidence elucidated the molecular mechanisms of the proliferation- and tumourigenesis-promoting function of the signalling pathway and suggested that the signalling pathway may provide potential candidates for prognostic markers and therapeutic targets.

## Results

### Upregulation of ANRIL correlates with nasopharyngeal carcinoma progression

**Figure 1:**
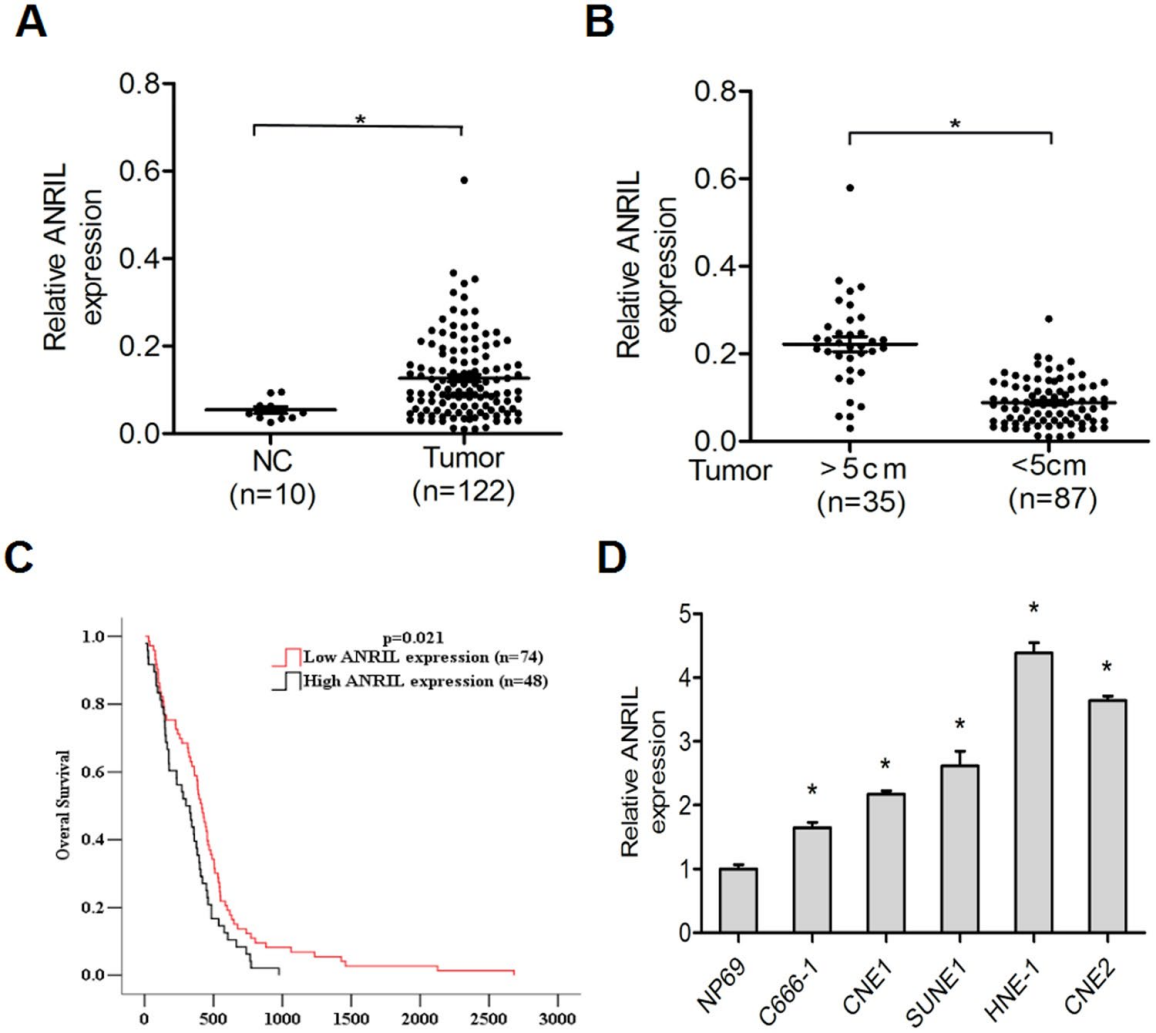
Upregulation of ANRIL expression correlates with nasopharyngeal carcinoma progression. (**A**) Relative ratios of ANRIL expression in 122 nasopharyngeal carcinoma tissues and 10 normal nasopharyngeal tissues. (**B**) Comparison of ANRIL expression in 35 nasopharyngeal carcinoma tissues (tumour > 5 cm) and 87 nasopharyngeal carcinoma tissues (tumour < 5 cm). (**C**) Analysis of overall survival in patients with low or high ANRIL expression levels. (**D**) Histograms of the average relative expression of ANRIL in five nasopharyngeal carcinoma cell lines (C666-1, SUNE-1, CNE1, CNE2 and HNE-1) and the normal nasopharyngeal cell line NP69 by quantitative real-time RT-PCR analyses. Data are presented as the mean ± SD. *P < 0.05. In A, B and D, data are representative of two or three independent experiments with similar results.

To determine the expression of ANRIL in nasopharyngeal carcinoma, qRT-PCR analysis was performed in 122 nasopharyngeal carcinoma tissue samples and 10 normal nasopharyngeal tissue samples. The results showed that ANRIL expression was higher in nasopharyngeal carcinoma tissues compared with normal nasopharyngeal tissues (Fig. 1A). Furthermore, we next measured the ANRIL expression levels in tumour tissue samples with different clinicopathological characteristics, and the result implied that the ANRIL expression level was positively correlated with tumour size (Fig. 1B). Moreover, to evaluate the correlation between ANRIL expression and nasopharyngeal carcinoma patient prognosis, Kaplan-Meier survival analysis was performed. The curve indicated that patients with high ANRIL expression had a shorter survival times (Fig. 1C).

We next investigated ANRIL expression in five nasopharyngeal carcinoma cell lines (C666-1, SUNE-1, CNE1, CNE2 and HNE-1) and a normal nasopharyngeal cell line, NP69, by qRT-PCR and found higher ANRIL expression in C666-1, SUNE-1, CNE1, CNE2 and HNE-1 cells than in NP69 (Fig. 1D). These results suggested that ANRIL is associated with the progression of nasopharyngeal carcinoma.

### Depletion of ANRIL inhibits nasopharyngeal carcinoma proliferation and tumourigenesis

**Figure 2:**
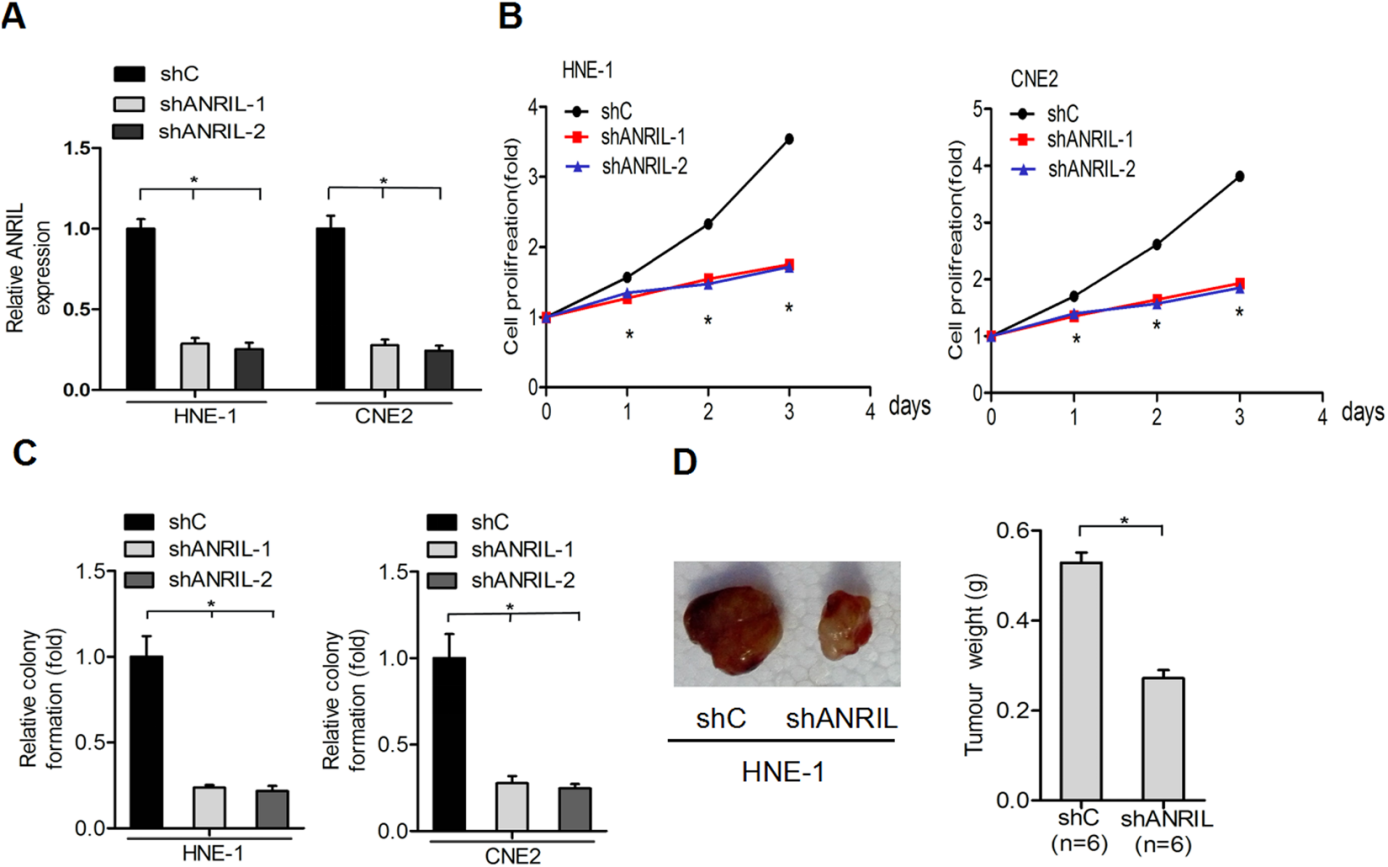
Depletion of ANRIL inhibits nasopharyngeal carcinoma proliferation and tumourigenesis. (**A**) QRT-PCR analyses were used to analyse ANRIL expression after stable knockdown in HNE-1 and CNE2. The proliferation (**B**) and colony formation (**C**) of HNE-1 and CNE2 cells were analysed after stable ANRIL depletion. (**D**) The effects of shANRIL on subcutaneous tumour generation (n = 6) in the HNE-1 cell line. Data are presented as the mean ± SD. *P < 0.05. In A, B, C and D, data are representative of two or three independent experiments with similar results.

It has been reported that the overexpression of ANRIL is a common event in several types of malignant tumour and that the abnormal elevation of ANRIL is associated with the enhancement of tumour proliferative ability. To determine whether ANRIL promotes an oncogenic phenotype of nasopharyngeal carcinoma, lentivirus-mediated short hairpin RNAs (shRNAs) targeting ANRIL or a non-silencing control were used in HNE-1 and CNE2 cells (Fig. 2A). As shown in Fig. 2B and C, shRNA knockdown of endogenous ANRIL markedly inhibited the proliferation of HNE-1 and CNE2 cells compared with the control. ANRIL knockdown also impaired colony formation in both nasopharyngeal carcinoma cell lines.

Consistent with the *in vitro* observations, the xenograft tumours formed in the sh-ANRIL group were generally smaller than those in the control group. The tumour growth of the sh-ANRIL group was significantly slower than that of the control group (Fig. 2D). Collectively, our data suggest that ANRIL is an important contributor to nasopharyngeal carcinoma proliferation and tumourigenesis.

### SOX2 induces the expression of ANRIL by promoting its transcription

**Figure 3:**
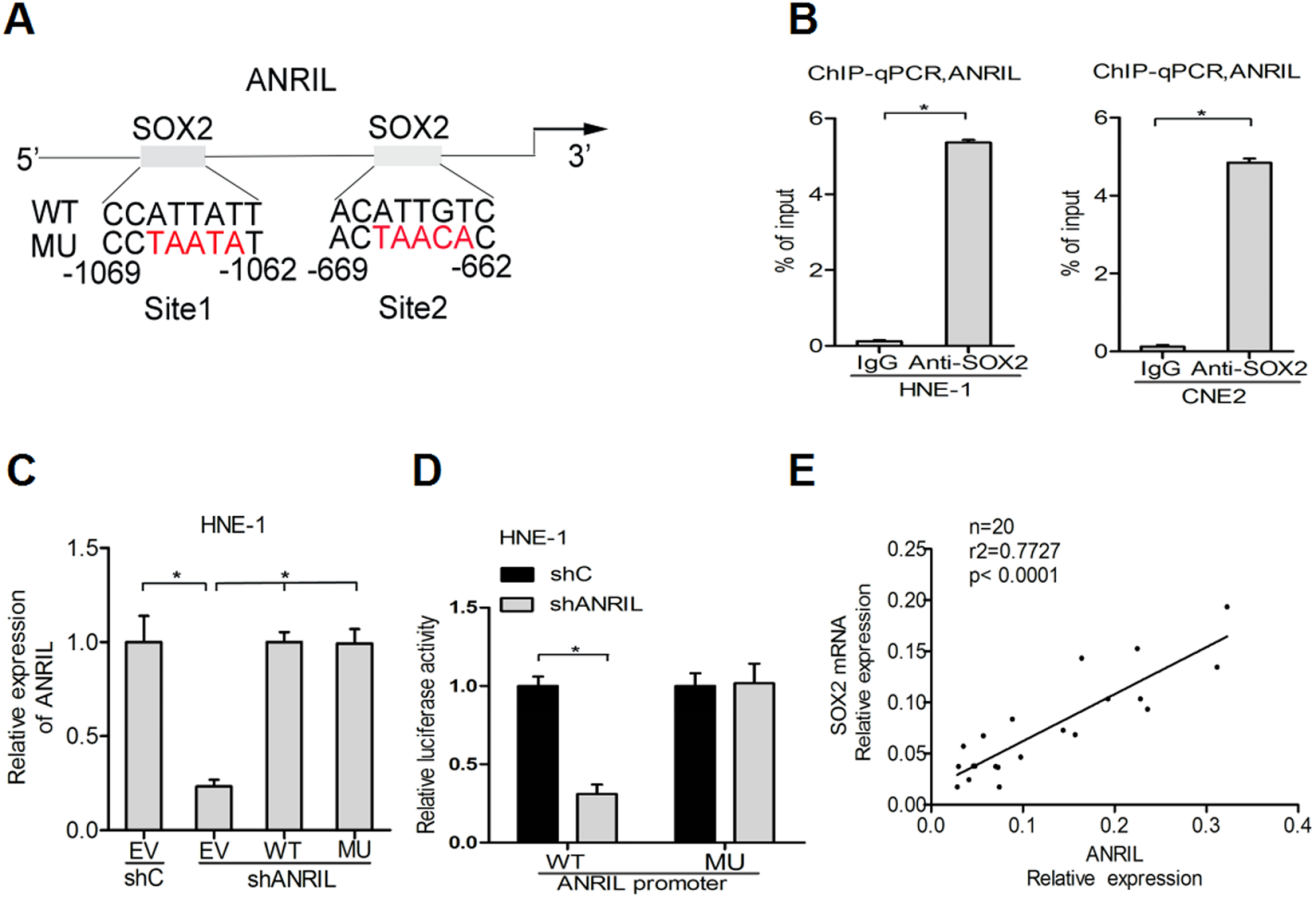
SOX2 induces the expression of ANRIL by promoting its transcription. (**A**) Bioinformatics analysis of the binding site of SOX2 on the ANRIL promoter. ChIP-qPCR (**B**) and dual luciferase assay (**C**) were used to confirm the binding of SOX2 with the ANRIL promoter. (**D**) Quantitative real-time RT-PCR analysed the correlation of ANRIL and SOX2 transcripts. Data are presented as the mean ± SD. *P < 0.05. In B and C, data are representative of two or three independent experiments with similar results.

Recently, it has been reported that many transcriptional factors may be involved in regulating lncRNA transcription. To investigate which transcriptional factors can interact with ANRIL and induce its expression, one open-access database was used to analyse the potential binding sites of transcriptional factors in the promoter region of ANRIL (http://jaspar.genereg.net/). SOX2 was the predicted TF, with one binding site on the ANRIL promoter (Fig. 3A). To test whether SOX2 could bind directly to the promoter region and activate the transcription of ANRIL, chromatin immuno-precipitation (ChIP) was carried out in two nasopharyngeal carcinoma cell lines, HNE-1 and CNE2, using antibodies against SOX2. The results in the two nasopharyngeal carcinoma lines showed that the complexes immunoprecipitated by the two anti-SOX2 antibodies were enriched in the ANRIL promoter DNA fragment, compared with the isotype antibody control. In addition, the ChIP-derived DNA fragment complexes were amplified by quantitative PCR in the two nasopharyngeal carcinoma lines (Fig. 3B). Increased SOX2-binding activity on the ANRIL promoter was observed by dual luciferase reporter assays (Fig. 3C). Next, the correlation assay was performed between the expression of ANRIL and SOX2 in 20 nasopharyngeal carcinoma tissues. Using qRT-PCR, a statistically significant correlation was observed between ANRIL levels and SOX2 transcript levels (r^**2**^ = 0.7727, p < 0.001; Fig. 3D). In conclusion, SOX2 induces the transcription of ANRIL.

### ANRIL is required for SOX2-driven nasopharyngeal carcinoma proliferation

**Figure 4:**
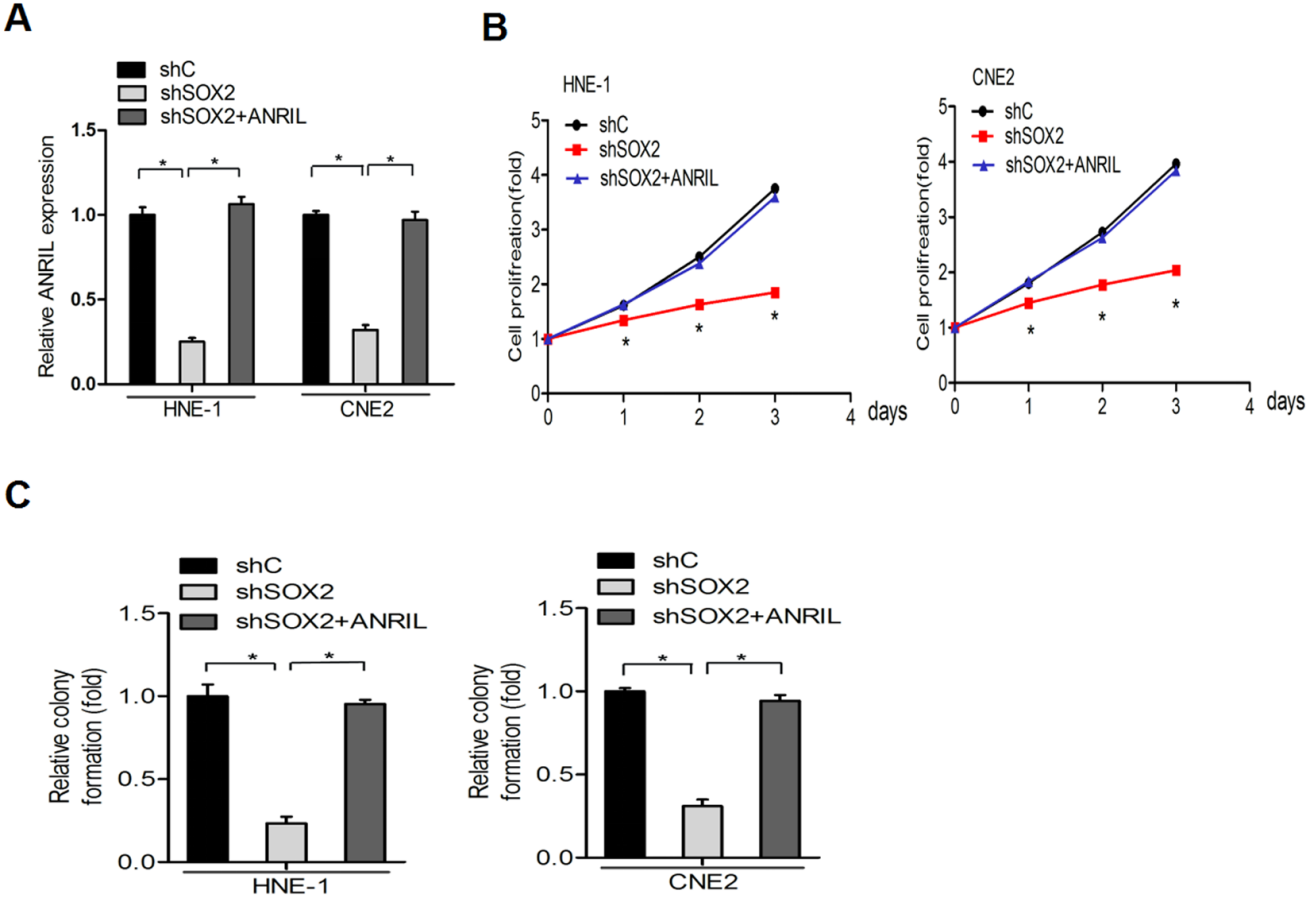
ANRIL is required for SOX2-driven nasopharyngeal carcinoma proliferation. (**A**) ANRIL expression levels in HNE-1 and CNE2 cells transfected with SOX2 shRNAs alone or in combination with the ANRIL vector were analysed by quantitative real-time RT-PCR assays. Cell growth (**B**) and colony formation (**C**) were analysed in HNE-1 and CNE2 with SOX2 shRNAs alone or in combination with ANRIL. Data are presented as the mean ± SD. *P < 0.05. In A, B and C, data are representative of two or three independent experiments with similar results.

To further investigate whether ANRIL overexpression could enhance proliferation in SOX2-depleted cells. An ANRIL vector was ectopically expressed, and the overexpression efficiency was measured in HNE-1 and CNE2 nasopharyngeal carcinoma cells. As shown in Fig. 4A, the overexpression of ANRIL compensated for the expression of ANRIL that had been suppressed by SOX2 knockdown. ANRIL could rescue the suppressive effects of SOX2 knockdown on nasopharyngeal carcinoma cell proliferation (Fig. 4B and C). Collectively, these results indicate that ANRIL is critical for SOX2-induced nasopharyngeal carcinoma growth.

### ANRIL/β-catenin is essential for SOX2-mediated nasopharyngeal carcinoma progression

**Figure 5:**
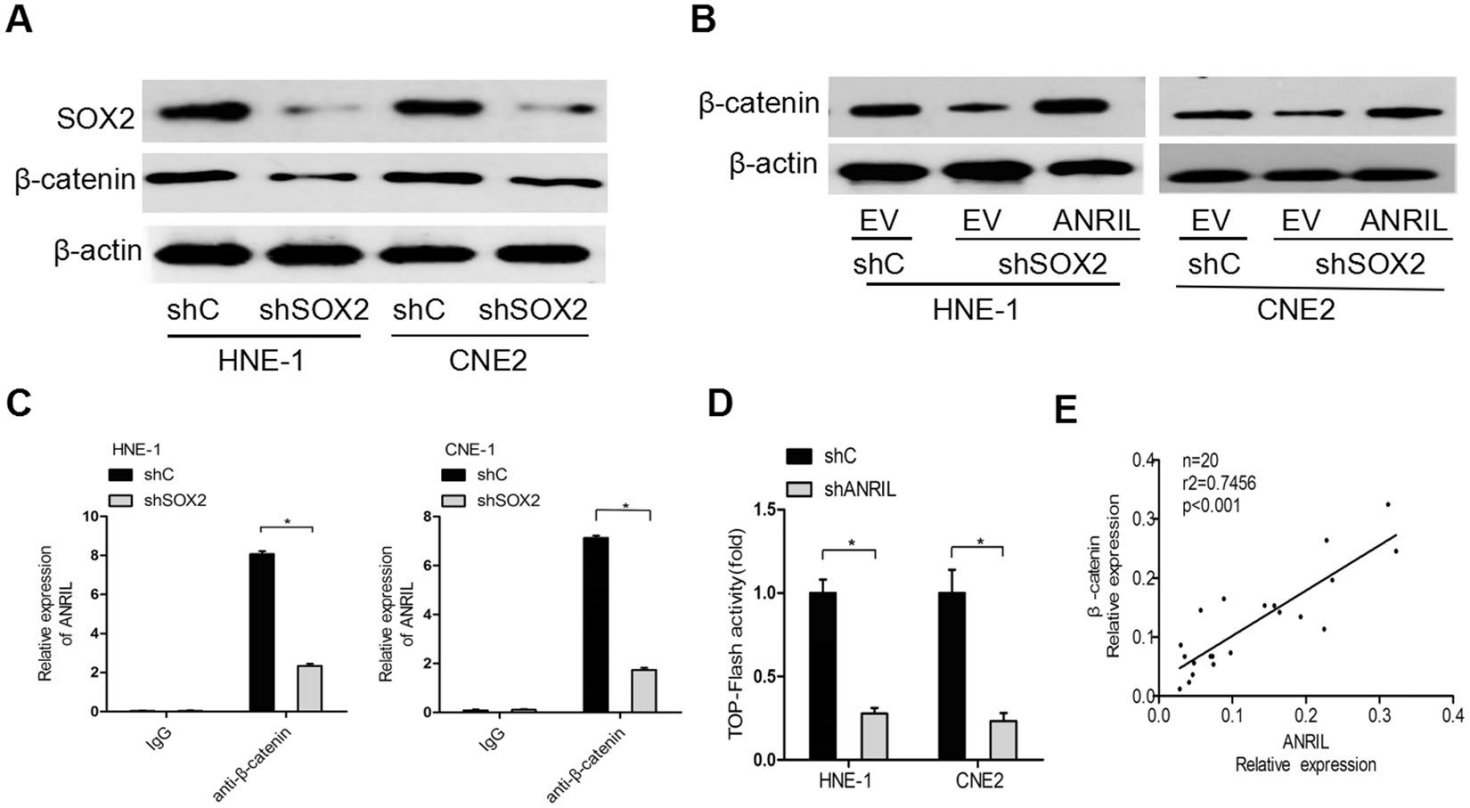
ANRIL/β-catenin is essential for SOX2-mediated nasopharyngeal carcinoma progression. (**A**) Levels of SOX2 and β-catenin protein expression in HNE-1 and CNE2 after stably sh-SOX2 and shControl. (**B**) β-catenin protein expression analysis in HNE-1 and CNE2 with SOX2 shRNAs alone or in combination with ANRIL vector. (**C**) Schematic diagram indicated the β-catenin binding to ANRIL region by RNA IP assays. (**D**) TOP-FOP flash assay was performed to evaluate the effect of shANRIL on WNT/β-catenin pathway. (**E**) Correlation between ANRIL and β-catenin transcript determined by quantitative real-time RT-PCR in 20 human nasopharyngeal carcinoma specimens.

Since β-catenin is an important downstream effector of ANRIL in cancer^22^, we assessed whether ANRIL mediates β-catenin expression in nasopharyngeal carcinoma cells. We first demonstrated that the depletion of SOX2 could suppress β-catenin expression (Fig. 5A). Overexpression of ANRIL markedly restored SOX2 knockdown-inhibited β-catenin expression in HNE-1 and CNE2 cells (Fig. 5B). Furthermore, we demonstrated by RIP assays that ANRIL could bind to β-catenin. The result was consistent in HNE-1 and CNE2 cells, which both showed a reduction of the formation of the ANRIL and β-catenin complex due to SOX2 knockdown (Fig. 5C). The knockdown of ANRIL resulted in decreased TOP-Flash reporter gene activity (Fig. 5D). These data suggest that SOX2 mediates ANRIL mRNA expression to regulate β-catenin expression.

To further assess the clinical relevance of our findings in this study, we examined the expression of ANRIL and β-catenin in clinical nasopharyngeal carcinoma samples. We collected 20 clinical snap-frozen nasopharyngeal carcinoma samples and performed qRT-PCR analyses. As shown in Fig. 5E, based on the quantification of mRNA expression, ANRIL was revealed to be significantly correlated with β-catenin according to Spearman’s rank correlation analysis (r^2^ = 0.7456, p < 0.0012). In conclusion, the activation of the WNT/β-catenin pathway is mediated by the SOX2-ANRIL pathway in nasopharyngeal carcinoma.

## Discussion

Increasing evidence indicates that the abnormal expression of lncRNAs is critical for the development of the malignant phenotype of nasopharyngeal carcinoma cells^23,24^. In this study, we demonstrated that ANRIL promotes nasopharyngeal carcinoma growth and tumourigenesis. Furthermore, we confirmed the clinical relevance of ANRIL in human nasopharyngeal carcinoma tissues. In conclusion, we ascertained that ANRIL functions as a regulator of nasopharyngeal carcinoma progression.

The molecular mechanisms of ANRIL regulation of cell proliferation are not well understood. Recent studies have shown that ANRIL promotes the invasion and metastasis of thyroid cancer by suppressing p15INK4B expression through the TGF-β/Smad signalling pathway^25^. In the present work, we confirm that ANRIL specifically binds to β-catenin and increases the transcriptional activity of the WNT/β-catenin pathway. The nuclear β-catenin associated with the T cell factor (TCF)/ lymphoid enhancer factor (LEF) family of transcription factors plays roles in several compounds including cyclin D1 and c-myc, which could act as a tumour activator in colorectal cancer^26^. Moreover, a recent study reported that increasing ZNF191 binding to the β-catenin promoter resulted in inducing the transcription of β-catenin in hepatocellular carcinoma^27^. Another study confirmed that KLF5 knockdown inhibited β-catenin/TCF transcriptional activity in colon cancer cells^28^. Let-7a directly binds to ANRIL in nasopharyngeal carcinoma^29^. In this study, we found that ANRIL plays important roles in nasopharyngeal carcinoma growth and tumourigenesis. We provide strong evidence that SOX2 directly binds to the ANRIL promoter and is required for ANRIL transcription. In addition, inhibition of SOX2 by shRNA strongly inhibits ANRIL expression, indicating that SOX2 and ANRIL are required for nasopharyngeal carcinoma proliferation and tumourigenesis. We confirmed that SOX2 functions as a critical molecule in the regulation of human nasopharyngeal carcinoma. In agreement with the results of a previous report, we found that SOX2 could be a marker of poor clinical outcomes in nasopharyngeal carcinoma^30^. Another previous study demonstrated that the upregulation of SOX2-activated LncRNA PVT1 expression promotes breast cancer cell growth and invasion^31^. However, the relationship of ANRIL and β-catenin in nasopharyngeal carcinoma has not been extensively studied. In our study, we found that ANRIL and the β-catenin protein are positively correlated in human nasopharyngeal carcinoma tissues. We also observed that ANRIL regulates β-catenin expression by directly binding to β-catenin. Taken together, these results will help elucidate the role of ANRIL in promoting nasopharyngeal carcinoma progression.

In summary, SOX2 promotes the ANRIL-β-catenin pathway, leading to nasopharyngeal carcinoma growth. Our study indicates that the SOX2-ANRIL-β-catenin pathway may provide new opportunities for the development of therapeutic agents in human nasopharyngeal carcinoma.

## Materials and Methods

### Clinical tissue samples

In total, 122 nasopharyngeal carcinoma tissues and 10 normal nasopharyngeal tissues were collected from our department (Meizhou People’s Hospital) between January 2015 and December 2016. None of the patients had received chemotherapy and/or radiotherapy before the operation. To use the clinical materials for research, prior patient consent and approval from the Institutional Research Ethics Committee of the Meizhou People’s Hospital were obtained. Follow-up information was available for all patients. The use of tissue specimens was carried out in accordance with the approved guidelines of the Meizhou People’s Hospital. Written informed consent was obtained from each patient, and all patients granted permission for the data obtained to be used in subsequent studies.

### Cell lines

Five NPC cell lines (C666-1, SUNE-1, CNE1, CNE2 and HNE-1) and the normal nasopharyngeal cell line NP69 were purchased from the Cell Bank of the Chinese Academy of Sciences (Shanghai, China). All cells were maintained in DMEM, and other cells were cultured in RPMI1640 (GIBCO, Grand Island, NY, USA) supplemented with 10% FBS. Cells were cultured in a humidified incubator in a 5% CO_2_ atmosphere at 37 °C.

### Chromatin immunoprecipitation

Chromatin immunoprecipitation (ChIP) was performed using the Chromatin Immunoprecipitation kit (Upstate) according to the manufacturer’s instruction. Briefly, HNE-1 and CNE2 cells were treated with 1% formaldehyde to cross-link the proteins and DNA. The cell lysates were sonicated to shear DNA to sizes of 300 to 1000 bp. Equal aliquots of chromatin supernatants, into which 1 μg of either anti–SOX2 (Abcam) or anti-IgG as a negative control was added, were incubated overnight at 4 °C with rocking. After reverse cross-linking of the protein/DNA complexes to free the DNA, PCR was performed using specific primers to amplify a 230-bp region (site 1) of the β-catenin promoter (primers: forward 5′-GTGGTATTTTGAATTAAATTG-3′ and reverse 5′-CCCCGTCCCTTCCCTACTGG-3′) and a 278-bp region (site 2) of the β-catenin promoter (primers: forward 5′-GCCATAAAGCCTAGGGCATC-3′ and reverse 5′-TTACTATAAGCTTTAGGTC-3′).

### Luciferase promoter assay

Cells were seeded in triplicate in 48-well plates and allowed to settle for 24 hours. The pGL3-ANRIL promoter (100 ng) or the control luciferase plasmid plus 1 ng of pRL-TK Renilla plasmid (Promega) were transfected into HNE-1 cells using the Herrif TransTM Liposomal Transfection Reagent (Yi sheng, Shanghai, China) according to the manufacturer’s recommendations. Luciferase and Renilla signals were measured 48 hours after transfection using the Dual-Luciferase Reporter Assay kit (Promega) according to a protocol provided by the manufacturer. Three independent experiments were performed, and the data are presented as the mean ± SD.

### RNA extraction and quantitative RT-PCR

Total RNA was isolated using the Trizol reagent (Invitrogen). First-strand cDNA was generated using the PrimeScript® 1st Strand cDNA Synthesis Kit (TaKaRa, Dalian, China) and either gene-specific primers or random primers. Real-time PCR was performed on the StepOne™ Real-Time PCR System (Applied Biosystems, Foster City, USA) using FastStart Universal SYBR Green Master (ROX) (Roche). β-actin was employed as endogenous control. The primers for ANRIL were: 5′-TGCTCTATCCGCCAATCAGG-3′ and 5′-GGGCCTCAGTG GCACATACC-3′. The primers for β-catenin were: 5′-TTGAAAATCCAGCGTGGACA-3′ and 5′-TCGAGTCATTGCATACTGTC-3′. The primers for SOX2 were: 5′-CTCGTGCAGTTCTAC TCGTCG-3′ and 5′-AGCTCTCGGTCAGGTCCTTT-3′. The primers for β-actin were: 5′-CAT GTACGTTGCTATCCAGGC-3′ and 5′-CTCCTTAATGTCACGCACGAT-3′.

### Cell proliferation and colony formation

The cell proliferation assay was performed using a WST-1 Assay Kit (BioVision, Inc., Milpitas, CA, USA). A total of 400 HNE-1 and CNE2 cells were plated onto 6-well plates and incubated at 37 °C in a 5% CO_2_ incubator for 2 weeks. Fresh medium was added every 3 days. At the end-point, the cells were washed twice with cold phosphate-buffered saline (PBS), fixed with 4% paraformaldehyde for 30 minutes, and stained with 1% crystal violet solution for 20 minutes at room temperature. The visible colony numbers were counted. This experiment was performed in triplicate.

### Construction of vectors

The cDNA encoding ANRIL was PCR-amplified by Q5 High-Fidelity DNA Polymerase (BioLabs) and subcloned into the EcoR1 and Xho1 sites of the pcDNA3 vector (Invitrogen), subsequently named pCDNA3-ANRIL. pLVX-ANRIL was generated from pCDNA3-ANRIL.

### Construction of stable cell lines with overexpression or downregulation of ANRIL and SOX2

To obtain cell lines stably expressing ANRIL and SOX2, HNE-1 and CNE2 cells were transfected with the plasmids pLVX-ANRIL and pLVX-SOX2. The stably overexpressing cell lines were identified using real-time PCR and Western blot. To obtain cell lines stably suppressing ANRIL and SOX2, HNE-1 and CNE2 cells were transfected with the plasmids pLentilox 3.7-ANRIL and pLentilox 3.7-SOX2. The design of the shRNAs was assisted by web-based software provided by Invitrogen (http://rnaidesigner.invitrogen.com/rnaiexpress/).

### Western blot analysis

Cells were lysed in RIPA buffer, agitated for 20 minutes at 4 °C, sonicated for 15 seconds using a sonic oscillator and centrifuged at 12,000 rpm for 15 minutes. The total protein concentration was determined using the BCA method. Equal amounts of the total protein (30 µg) were then denatured and loaded on 10% SDS polyacrylamide gels for separation. The proteins were transferred onto polyvinylidene difluoride membranes that were subsequently blocked with 8% non-fat milk in TBST. The membranes were incubated with primary antibodies including SOX2 (Abcam) and β-catenin (1:1000, Cell Signaling Technology) at 4 °C overnight. β-actin (1:5000, Protech) was used as the loading control. After washing, the membranes were incubated with HRP-conjugated goat anti-rabbit, goat anti-mouse (Cell Signalling Technology, dilution 1:5000) secondary antibodies for 1 h at room temperature and visualized with an enhanced chemiluminescence detection kit (Millipore).

### RNA immunoprecipitation (RIP) assay

The cells were used to perform RNA immunoprecipitation (RIP) experiments using a β-catenin antibody (Abcam) and the Magna RIP™ RNA-Binding Protein Immunoprecipitation Kit (Millipore, Bedford, MA) according to the manufacturer’s instructions. The cells were lysed in complete RIP lysis buffer. A total of 100 μl of whole cell extract was incubated with RIP buffer containing protein G-agarose beads conjugated with antibodies against β-catenin or control IgG antibodies (Millipore) for 6 h at 4 °C. The beads were washed with wash buffer, and then the complexes were incubated with 0.1% SDS/0.5 mg/ml Proteinase K (30 min at 55 °C) to remove the proteins. The RNA concentration was measured using a NanoDrop spectrophotometer (Thermo Scientific), and its quality was assessed using a bioanalyser (Agilent, Santa Clara, CA). Finally, the immunoprecipitated RNA was purified and analysed by qRT-PCR.

### Xenografts

Female BALB/c-nude mice (4–5 weeks old and weighing 15–18 g) were housed under pathogen-free conditions. HNE-1 cells (2 × 10^6^) were trypsinized, washed twice with serum-free medium, reconstituted in serum-free medium DMEM, mixed 1:1 with Matrigel (Becton-Dickinson) and then inoculated subcutaneously into the right flank of each nude mouse. Tumour size was measured every 3 days with a digital calliper, and the tumour volume was calculated according to the formula: tumour volume (mm^3^) = length × width^2^ × 0.5. At the end of the experiment, all mice were sacrificed, and the total weights, tumour weights, and tumour volumes were recorded. All experimental procedures were carried out in accordance with the guidelines of and were approved by the Guidance of Institutional Animal Care and Use Committee of the Meizhou People’s Hospital.

### Statistical analysis

Statistical analyses were performed in GraphPad Prism version 5.0 for Windows (GraphPad Software Inc., San Diego, CA, USA). The significance of the data from patient specimens was determined by Pearson’s correlation coefficient. The significance of the *in vitro* and *in vivo* data between experimental groups was determined by Student’s t test or Mann-Whitney U-test. p < 0.05 was statistically significant.
